# Risk Modifying Factors of Anxiety and Depressive Disorders, Using the Example of a Population Study in the Żywiec District

**DOI:** 10.3390/ijerph181910248

**Published:** 2021-09-29

**Authors:** Bogumiła Lubecka, Marek Lubecki, Janusz Kasperczyk, Jadwiga Jośko-Ochojska, Robert Pudlo

**Affiliations:** 1 Individual Specialist Medical Practice Bogumiła Lubecka, 40-507 Katowice, Poland; 2 Individual Specialist Medical Practice Marek Lubecki, 40-507 Katowice, Poland; marek.lubecki@gmail.com; 3 Department of Environmental Medicine and Epidemiology, Medical University of Silesia, 41-808 Zabrze-Rokitnica, Poland; jkasperczyk@sum.edu.pl (J.K.); jjosko@sum.edu.pl (J.J.-O.); 4 Department of Psychiatry, Medical University of Silesia, 42-612 Tarnowskie Góry, Poland; rpudlo@sum.edu.pl

**Keywords:** anxiety disorders, depression, population-based study, risk factors

## Abstract

The aim of this study was the identification of the risk modifying factors of anxiety and depressive disorders based on a population study. This study was conducted in a randomly selected group of 1659 adult inhabitants of the Żywiec district. Anonymous questionnaires consisting of a proprietary questionnaire and the Hospital Anxiety and Depression Scale (HADS) were used to collect the data. The conducted analysis revealed that the factors increasing the risk of depressive disorders in the studied population were female gender, age over 60, retirement period, primary and vocational education, unemployment, mental work and absolute lack of physical activity, but also daily and intensive sports, heavy smoking, chronic somatic diseases and misuse of sleeping pills and over-the-counter sedatives. Anxiety disorders occurred more often in the group of unemployed, self-employed or retired people. They also occurred more often in the group of people who do not perform any physical activity and use alcohol every day, but also among those who maintain abstinence, regularly smoke tobacco and use stimulants, suffer from somatic diseases and overuse sleeping drugs. Disease preventive factors for anxiety disorders and depression were a constant form of employment, moderate and regular physical activity, avoiding the use of psychoactive substances and the regular treatment of comorbid somatic diseases and insomnia.

## 1. Introduction

Mental disorders’ prevalence rates have increased significantly in recent years. The most common ones are anxiety disorders and depression. According to WHO (World Mental Health) data, depression accounts for over 4% of the global burden of all diseases and is the second most common cause of disability after cardiovascular disease [1]. Anxiety disorders can be considered as a separate nosological unit or component of the depressive episode. These disorders are often comorbid, as anxiety is a risk factor for depression and vice versa [2].

The most up-to-date data on the prevalence of these disorders in Poland are described in the EZOP Poland (Epidemiology of Psychiatric Disorders and Availability of Psychiatric Healthcare) research conducted in 2012. It showed that 23.4% of the respondents had been diagnosed with at least one mental disorder during their lifetime, of which 9.6% had symptoms of anxiety disorders and 3.4% met the criteria for an episode of major depression. These data only include the adult population below 65 years of age [3]. 

The European-wide ESEMeD study (The European Study of the Epidemiology of Mental Disorders), published in 2004, showed that major depression and specific phobias were among the most common mental disorders, which occurred with a frequency of 13% and 8%, respectively, during the lifetime of the respondents [4]. Nowadays, more and more importance is committed to the correct diagnosis and treatment of the described disorders. The current standards assume that thanks to properly conducted preventive measures, many new cases can be avoided. Prevention programs carried out in Poland so far only cover people belonging to high-risk groups, such as women after childbirth, adolescents and people over 65, which from the point of view of the increasing frequency of anxiety disorders and depression in the general population seems to be insufficient [5]. 

To create effective prevention programs, it is necessary to conduct many population studies, the effect of which will be to identify new and verify already studied risk factors for the occurrence of anxiety disorders and depression in individual populations. Apart from the aforementioned EZOP study, the population studies conducted in Poland so far are based on the analyses of less numerous groups selected for specific risk factors, such as school students [6], students [7,8], patients with another mental [9] or somatic disease [10,11,12] and the elderly [13,14].

This publication presents the analysis of the correlation between individual risk modifying factors and the occurrence of anxiety and depressive disorders in the adult population of the Żywiec district. The modifying factors were grouped in terms of increasing the risk of developing the disease or having a protective effect, which made it possible to distinguish other groups requiring special preventive measures.

## 2. Materials and Methods

The study population consisted of the inhabitants of the Żywiec district. This region is inhabited by 124,616 people, of which 64,149 are women and 60,467 men. Data were collected in the city of Żywiec, as well as in all regions of the district. Due to its location, compact geographic structure, organization of healthcare and demographic characteristics, the Żywiec district was considered optimal for obtaining reliable data for epidemiological research. In terms of socio-demographic factors, the studied group corresponded to the district population. Therefore, the collected data can be extrapolated to the Polish population. The obtained data were also used to assess the disproportion between the occurrence and treatment of the studied disorders, which was described in another publication. 

Data for the study were collected with anonymous questionnaires. The study group consisted of randomly selected adult inhabitants of the Żywiec district who voluntarily and independently completed the questionnaires. The inclusion criteria were: living in the district, being 18 years of age and giving informed consent to participate in the study. The exclusion criteria were as follows: uncorrected visual disturbances making it impossible to fill in the questionnaire, cognitive disorders that make filling in the questionnaire impossible and submitting an incomplete questionnaire or questionnaire containing interpretation doubts. In order to obtain a representative study group, activities were carried out in many different places (Poviat Starosty, City Hall, secondary vocational and high schools, family doctor clinics and multi-specialist clinics, among employees of hospitals and local institutions and companies). They included several ways of reaching residents: direct contact and handing over a questionnaire, making it possible to take questionnaires from prepared and marked places in public buildings, and handing over the questionnaires to people ready to distribute them among their families, friends and colleagues. The distributed questionnaires were collected in person from designated places.

A research questionnaire consisting of two parts was used for the study. The first part included questions about age, marital status, place of residence, professional activity, physical activity, the use of psychoactive substances, previously diagnosed somatic and mental illnesses and medications taken. In the second part, the Hospital Scale of Anxiety and Depression was used to diagnose studied disorders, which is an inventory of self-esteem. Due to the high sensitivity and specificity in detecting generalized anxiety and depressive disorders, this scale was also used in the diagnosis of healthy adults in over 700 scale world studies, showing satisfactory psychometric properties for both the Anxiety (HADS-A) and Depression (HADS-D) subscales. In this research, the Polish version of the scale was used. The use of this scale in research is supported by the short time needed for self-completion, clearly formulated questions (regardless of gender, age and level of education) and the possibility of simultaneous testing of both disorders. The Hospital Scale of Anxiety and Depression consists of two subscales with 7 questions each—HADS-D (Hospital Scale of Anxiety and Depression—Depression Subscale) and HADS-A (Hospital Scale of Anxiety and Depression—Anxiety Subscale)—sequentially for the study of depression and anxiety disorders. The total number of points obtained corresponds to the severity of the symptoms of anxiety and depression. Values from 0 to 7 points correspond to the norm, values from 8 to 10 points correspond to the limit value and values greater than or equal to 11 points represent an episode of depression (HADS-D) or an anxiety disorder (HADS-A) [15].

The correlation of the results obtained in the scales with the risk factors listed in the questionnaire was examined. Statistical analysis was performed using the Statistica 13.0 (Statsoft, Kraków, Poland) package and included: descriptive statistics (group sizes, percentage fractions for qualitative variables, mean for quantitative variables, standard deviation) and intergroup comparisons for quantitative variables, which were made using the t-test or analysis of variance—in the event of failure to meet the test assumptions, their non-parametric equivalents were used. For qualitative variables, the following tests were used: Pearson’s Chi2 and Maximum Likelihood. In the analyses, the *p*-value < 0.05 was considered significant.

This study is part of a larger project analyzing the epidemiology of depression, anxiety and sleep disorders in the mentioned region. The first part includes the assessment of the disproportion between the incidence of anxiety and depressive disorders and the use of psychiatric healthcare by the inhabitants of the district. It has been published already in Polish science magazine “Psychiatry” vol. 17, no. 1, 1–8 in 2020.

The research project was presented to the Bioethics Committee of the Medical University of Silesia, which concluded that the study did not require its consent. All respondents gave their informed consent to participate in the study.

## 3. Results

A total of 5000 questionnaires were distributed, 1659 of which were fully completed. Incomplete or incorrectly completed questionnaires were rejected. There were 934 women (56.3%) and 725 men (43.7%) in the group. The age of the respondents ranged from 18 to 92 years old. The mean age was 43.7 ± 14.61 years, and the median was 44 years. Three age groups were adopted: 18–39 years old, 40–59 years old and from 60 years old, representing 38.5%, 47% and 14.5% of the entire sample, respectively. The largest group in the study were people under 60 (85.5%). The subjects who were in a stable relationship prevailed (69.2%). Unmarried persons accounted for 30.8%. Due to the district’s demographic structure, inhabitants of communes predominated in the study (69.4%). Most respondents had secondary or vocational education (62%), and the most common form of employment was full-time work (62.9%). The characteristics of the study group in terms of selected risk modifying factors are presented in Table 1.

Physical activity was confirmed by 75.1% of the respondents, of which 6.9% performed it daily. Most of the respondents used alcohol sporadically or not at all. The use of stimulants was declared by: 19.8% who smoked cigarettes, 3.7% marijuana and derivatives and 1.3% psychostimulants. A total of 33.9% of the respondents confirmed the coexistence of somatic diseases, of which 6.5% were without regular treatment. Among the respondents, as many as 23.4% confirmed the use of over-the-counter calming supplements/medications and hypnotics, and 8.7% were treated for insomnia. 

**Figure 1:**
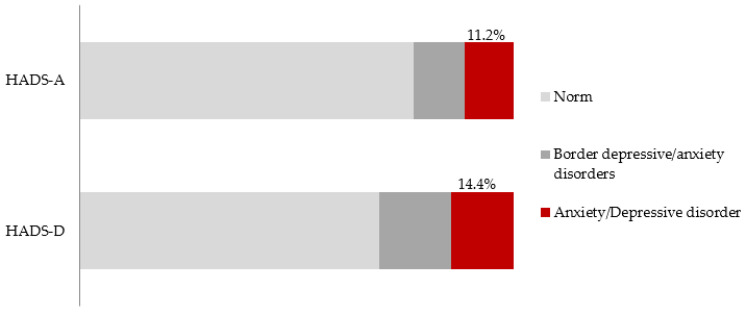
Results of the HADS-D and HADS-A subscales in the studied population.

In the study population, on the basis of the HADS results, depressive disorders were found in 14.4% of the respondents, and the criteria for anxiety disorders were met by 11.2% of the participants (Figure 1).

The examined risk factors were divided into groups. The first group included sociodemographic variables such as sex, age, marital status and place of residence. Detailed results obtained in the scale study are presented below (Table 2). To present the results, the study group was divided into subgroups depending on the result on the HADS scale. On the depression subscale (HADS-D), the sum of the points obtained by respondents from 0 to 7 points corresponds to the norm (HADS-D1), from 8 to 10 is the limit value of depression (HADS-D2) and a sum greater than or equal to 11 points represents an episode of depression (HADS-D3). On the anxiety subscales (HADS-A), the sum of points obtained during the study from 0 to 7 testifies to the norm (HADS-A1), a sum from 8 to 10 points is the limit value of an anxiety disorder (HADS-A2) and a sum greater than or equal to 11 points represents an anxiety disorder (HADS-A3).

**Figure 2:**
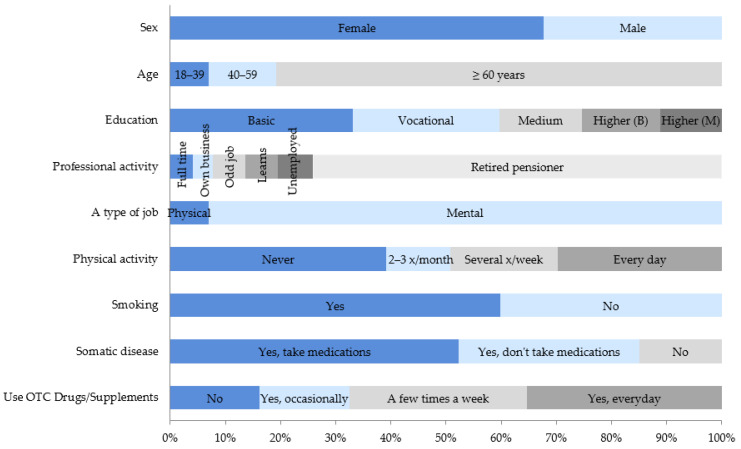
Factors modifying the risk of depressive disorders (HADS-D3) that were statistically significant.

**Figure 3:**
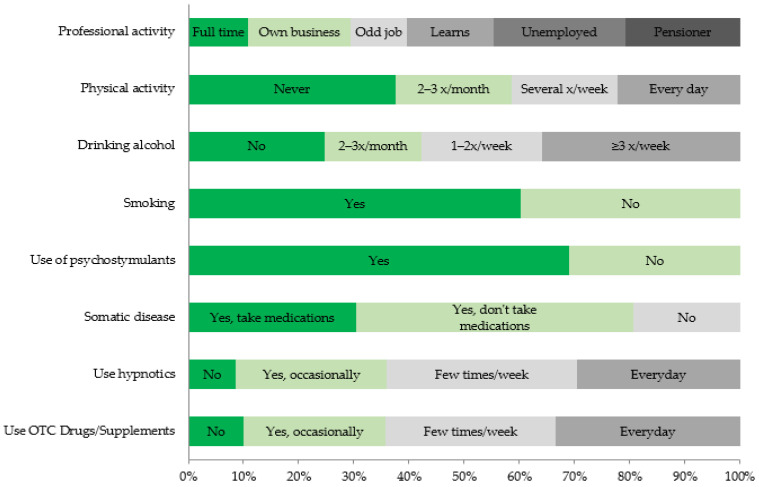
Factors modifying the risk of anxiety disorders (HADS-A3) that were statistically significant.

Modifying factors, the relationship of which with the occurrence of anxiety and depressive disorders was statistically significant, are presented in the diagrams (Figure 2 and Figure 3).

The prevalence of anxiety disorders in women and men was similar. Women met the criteria for depressive disorders significantly more often than men. In the group of respondents over 60 years of age, depressive disorders occurred much more often and were found in 34.9% of people compared to those in the age group 18–39 years old (5.7%) and 40–59 years old (3.3%). Anxiety disorders also slightly prevailed in this age group, but this was not a statistically significant difference. Marital status and place of residence did not significantly affect the occurrence of anxiety and depression.

**Figure 4:**
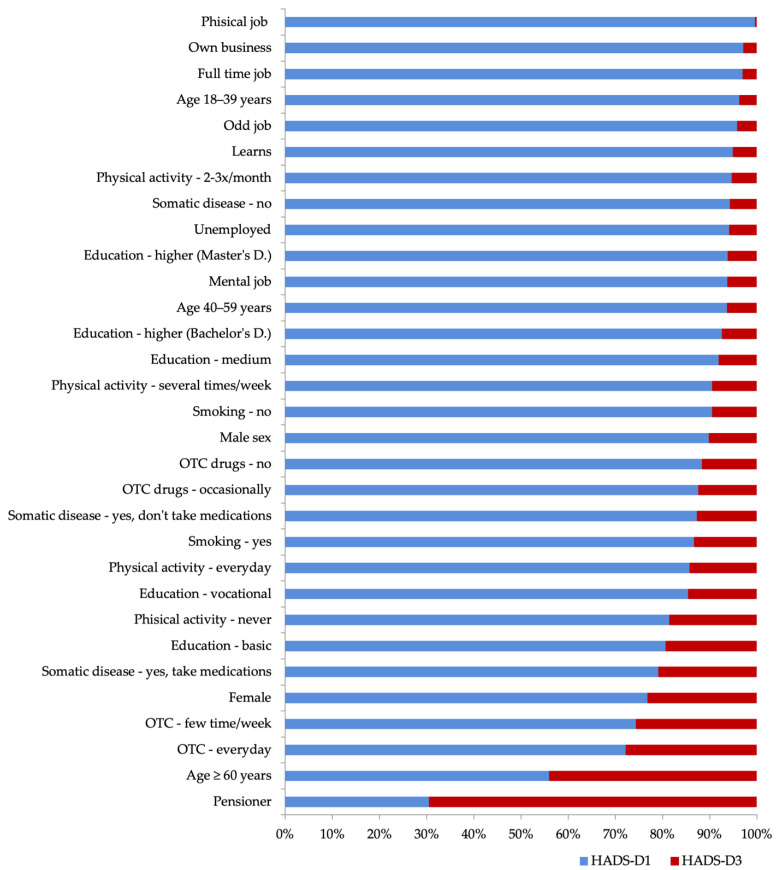
Data sorted by the percentage of depressive disorders (HADS-D3/HADS-D1) in particular subgroups of risk modifying factors that were statistically significant (the level of significance for all factors is *p* < 0.01).

**Figure 5:**
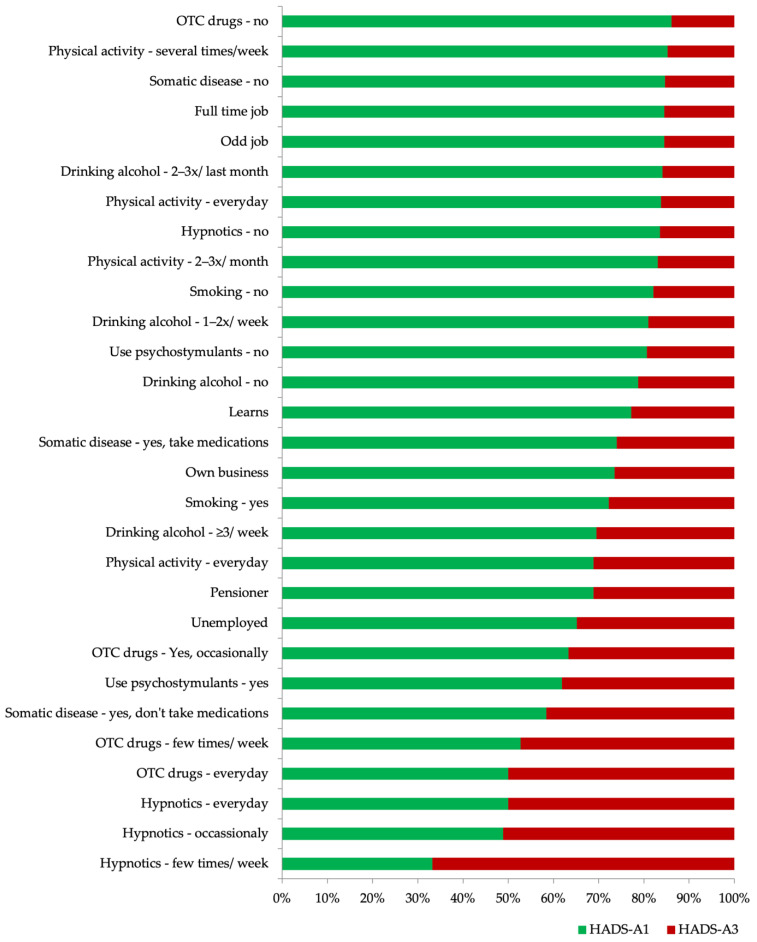
Data sorted by the percentage of anxiety disorders (HADS-A3 / HADS-A1) in particular subgroups of risk modifying factors that were statistically significant (the significance level for the effect of psychostimulants is *p* < 0.05; for other factors *p* < 0.01).

Data sorted by the percentage of the studied disorders depending on the individual subgroups of modifying factors, the relationship of which was statistically significant, are presented in Figure 4 and Figure 5. 

Most often, depressive disorders were presented by people with primary education (15.9%) and vocational education (12.8%). Compared to them, the incidence of depression was almost three times lower in the group of respondents with a master’s degree (5.4%). The level of education did not have a significant influence on the incidence of anxiety disorders.

The second group of analyzed factors concerned variables related to professional activity, type of work and physical activity. In the group of retired people, depressive disorders concerned over half of the respondents (51%). Compared to them, the incidence of depression in the unemployed, students or part-time workers was 4%, while in those working full-time or who are self-employed, it was 2%. The unemployed, retired and self-employed people were in the highest risk group of anxiety disorders. They occurred in this group twice as often as among full-time employees. A dozen times higher incidence of depression was observed in the respondents engaged in mental work than in the group of physical workers. The type of work was not significant for the occurrence of anxiety disorders.

In the studied population, the prevalence of anxiety and depression was significantly influenced by physical activity. In people who did not take up physical activity at all, anxiety disorders and depression occurred much more often than in other groups. However, in the group of people practicing sports every day, depressive disorders occurred slightly more often than among respondents who took care of physical activity less regularly.

The third group of the analyzed risk factors involved the use of psychoactive substances. The relationship between the frequency of alcohol consumption and the occurrence of anxiety disorders was confirmed, which was most often found in the group of people who drink alcohol three or more times a week (26.5%). It was observed that in the group of people who maintain abstinence, the frequency of anxiety disorders was slightly higher (18.2%) than among the sporadic drinkers. There was no relationship between the frequency of alcohol consumption and the occurrence of depressive disorders.

In the group of smokers, both anxiety disorders and depression were significantly more frequent. The use of marijuana was confirmed by a small group of people, a total of 62 respondents. A total of 75% of people in this group were under the age of 29. Among them, no statistically significant difference was found in the assessment of the prevalence of anxiety and depression. The use of psychostimulants was confirmed by 22 people. Respondents using stimulants almost twice as often presented symptoms of anxiety disorders. However, their use was not associated with a higher incidence of depression.

The fourth group of analyzed risk factors concerned variables related to the presence and the treatment of somatic diseases, the use of hypnotics, and thus the occurrence of insomnia, and the use of over-the-counter supplements or sedative agents. Among the subjects burdened with somatic diseases, anxiety disorders and depression were significantly more frequent than in healthy respondents. Depression was slightly more frequent (17.8%) in the group of patients receiving regular medical treatment for somatic diseases than in the group not taking regular medications (11.1%). Contrary to this observation, it was shown that somatically burdened people who did not receive regular treatment confirmed the occurrence of anxiety disorders significantly more often (34.3%) compared to people who regularly take medications (20.7%).

Depression was much more common in the group of people using sleeping pills than in respondents who refused to use them. This difference, probably due to the small size of the group, was not statistically significant. Anxiety disorders occurred significantly more often in subjects who used sleeping pills than in those untreated because of insomnia. Anxiety disorders and depression were significantly more frequent among respondents using herbal preparations or dietary supplements with a sedative effect compared to the rest of the studied population. In the group of people who confirmed the daily use of the above-mentioned substances, depressive disorders occurred twice as often and anxiety disorders were almost four times more frequent than in the population of people who refused to take them.

## 4. Discussion

Due to the significant social costs of anxiety and depression, it is important to identify risk factors and intervene early. Because of a small number of new national population studies on anxiety and depression in recent years, the authors planned and conducted a study of inhabitants of the Żywiec district, which was considered optimal for obtaining data for epidemiological research.

Due to the planned large size of the study group, the only possible method was a questionnaire study. The scale included in the survey that was used for the analysis was the Hospital Scale of Anxiety and Depression. The use of this scale is supported by the short time needed to complete it independently, clearly formulated questions—regardless of gender, age and level of education—and the possibility of the simultaneous examination of both disorders [16].

In the studied population, an episode of depression was found in 14.4% of the respondents, and the point criteria for anxiety disorders were met by 11.2% of the individuals. The results are higher than in the nationwide EZOP study from 2012, which revealed that 9.6% of people had symptoms of anxiety disorders, 0.4% met the criteria of minor depression and 3.4% had an episode of major depression [3]. The HADS-D subscale does not differentiate episode severity.

On the other hand, the results are consistent with the European ESEMeD studies, which showed that during the lifetime of the respondents depressive disorders occurred with a frequency of 14.0%, and anxiety disorders in 13.6% [4].

The first risk modifying factor tested was gender. The results obtained by the authors of this study show a similar frequency of anxiety disorders in both women and men. This phenomenon may result from the progressive process of standardizing or reversing social roles. There is an increase in the importance of women in the public and professional spheres, greater consent to showing weakness by men and more frequent use of medical help by men. Similar observations can be found in some international [17] and Polish studies [8].

The study showed that depressive disorders occur twice as often in the group of women than in men, which is confirmed by other publications [18,19]. In the EZOP study cited earlier, minor depression was equally common in both sexes, while major depression and anxiety disorders occurred twice as often in women as in men, and it was found that the differences in prevalence were greater in older age groups [3]. The reason for these differences can be explained among others through hormonal changes in the group of women related to the menstrual cycle, pregnancy, postpartum and menopause [20,21]. In a population study conducted in 2005–2007 in Poland, it was shown that postmenopausal women frequently deteriorate in their mental state—19.8% of them have symptoms of depressed mood, and 27% of increased irritability [22]. The analysis of the responses of women and men did not show any significant differences between them. This result proves that the HADS scale can be used without fear that the gender of the examined person will influence the obtained answers. Similar conclusions were the result of other Polish and foreign authors [16,23]

Age was the second researched risk modifying factor. Depressive disorders occurred much more often (34.9%) in the population of people over 60 than in other age groups. People between 18 and 39 years of age were in the intermediate risk range, and here depression was present at the level of 5.7%. The most numerous group and at the same time burdened with the lowest risk of these disorders were people between 40 and 59 years of age (3.3%). The most frequent occurrence of anxiety disorders was recorded among respondents over 60 years of age, but this difference was not statistically significant. Interpretations of the influence of age on the risk of depression may include both biological and psychological factors.

Early and middle adulthood is an extremely intense period in terms of the necessity to make key decisions regarding the development of an individual. Increasing social pressure, constant volatility and instability in terms of place of residence, work, career and interpersonal relations are conducive to the feeling of strong stress [24]. The simultaneous need to enter the labor market, gain financial independence, continue personal development and start a family can be overwhelming and may cause anxiety and depression to occur more frequently in this age group [25]. 

The stage after the age of 40 seems to be a period of relative financial stabilization, subsidence in life roles and the stability of somatic health.

Depressive disorders appear more and more frequently in the elderly with the emergence of many new psychological factors and somatic diseases. The factors contributing to depression include loneliness, a feeling of uselessness after retirement, suffering from somatic diseases and a reduction in psychophysical fitness and thus independence [26]. It should be noted that the oldest respondent in this study was 92 years old. Several analyses of the prevalence of depressive disorders in the elderly were carried out in the country. In the PolSenior study, the prevalence of depression increased with age and ranged from 20 to 33% [27], and in a study of inmates of care and treatment facilities in the Bielsko district conducted in the years 2008–2010, severe depression was found in 10.9% of respondents. The presence of depressive symptoms impairs the quality of life of the elderly and often worsens the prognosis of comorbidities [13]. It is known from the literature that the prevalence of anxiety disorders in the elderly is lower than among young people and ranges from 3.2% to 21.6% of the elderly population [28]. It is widely accepted that anxiety disorders develop most frequently in adolescence or early adulthood. Less than 1% of anxiety disorders develop in people over 65, and 90% in people under 40 [29].

In this study, no significant correlation was observed between marital status and place of residence, understood as living in a city or rural area by the respondents, and the presence of anxiety and depression.

The EZOP study shows that an important risk factor for anxiety and depressive disorders is the termination of a relationship with a close person, e.g., due to divorce or the death of a partner [3,30]. However, this does not mean that staying in a stable relationship is a guarantee of mental health. The quality of the relationship, which includes the scope of mutual support, is of great importance. Some studies show that people in long-term marriages or partnerships reported anxiety about their mental health slightly more often than others [31]. 

The phenomenon of the disappearance of obvious differences also applies to the lifestyle related to the place of residence. The stereotypical approach is that the inhabitants of rural areas far from the city noise, living closer to nature, eat healthier and spend more time in the fresh air. In the studies that analyzed the incidence of mental disorders among inhabitants of rural areas and cities, it was found that people living in cities obtained higher incidence rates due to anxiety disorders and depression [32]. On the other hand, it would seem that the rural community is characterized by lower health awareness, stronger fears of stigmatization due to psychiatric treatment and less access to specialized healthcare. In one of the Polish studies, in a population of 90 patients of the hospital in Świecie, it was proved that a lower level of education and living in rural areas were strong risk factors for a depressive episode [33]. The results of the research conducted by the authors of this publication support the ongoing process of rural urbanization. This consists of abandoning the rural lifestyle, taking up employment within cities and increasing health awareness and the availability of medical care [34]. One should not forget about the more and more common phenomenon of willingly inhabiting rural areas by inhabitants of large cities. These situations more and more often lead to the similarity of rural and urban environments, also in terms of the spread of health phenomena.

In this study, people with primary and vocational education presented depressive disorders three times more often than in the group with higher education. In the group of respondents with secondary education, a slightly higher incidence of depression was observed than in people with higher education. There was no significant relationship between the occurrence of anxiety disorders and the level of education.

A lower level of education may be associated with insufficient knowledge about a healthy lifestyle, including the proper mechanisms of stress reduction, a healthy diet, attention to physical activity and adherence to the principles of sleep hygiene [35]. The relationship between the higher incidence of depression in people with lower education is also confirmed by the results of studies conducted by other authors [36]. 

It was observed that the protective factor for the occurrence of depressive disorders is a permanent form of employment, such as full-time work and own activity, where the incidence of depressive symptoms was found to be 2.8% and 2.5%, respectively. A slightly higher percentage of these disorders was presented by people performing odd jobs, people studying and the unemployed—between 4.0 and 4.4%. The greatest morbidity due to depression (51%) was found in the group of retirees and disability pensioners.

Among the factors affecting mental health analyzed under the EZOP project and studied by the Public Opinion Research Center in 2012, one of the most important factors was employment, understood as a source of income, but also social support [3,37]. People who lose their jobs have a much higher risk of developing depression [38]. On the other hand, the appearance of a mental illness in professionally active people negatively affects their functioning at work [39]. 

Due to the obtained results, one should consider such a significant difference in the incidence of depression among the unemployed and retirees and pensioners. It seems that a significant difference between the two forms of economic inactivity is the sense of irreversibility accompanying the transition to a disability or retirement pension. The retirement period, often identified with the beginning of old age, a sense of uselessness, a lack of motivation to act, inability to use free time and the deterioration of somatic health, is a strong depressant factor [40].

In the study, the most important risk factor for anxiety disorders was unemployment (28.9%). Retirement/disability pension (25.2%) and own activity (22.5%) are in second and third place. A slightly lower percentage of anxiety symptoms affects people in education (19%), and the lowest among respondents employed full-time (13.1%) and part-time workers (12.3%). One of the elements of social security is social insurance. Research shows that unemployed people are characterized by worse health than professionally active people. Psychosomatic symptoms of anxiety, free-flowing anxiety and insomnia are more common in them [41]. 

The analysis revealed a significant protective effect of physical work on the prevalence of depressive disorders. Mentally working people were several times more likely to develop symptoms of depression. The type of work did not affect the occurrence of anxiety disorders.

The impact of physical work can be compared to the effect of physical activity, the significant impact of which on the occurrence of depression and anxiety disorders was noted in the analyses. The subjects who did not engage in any activity had a much higher risk of both disorders. The lowest risk was for people who were active several times a week, but not every day. The studies conducted so far show that systematic physical activity has a protective effect against the occurrence of anxiety and depressive disorders [42,43]. 

Interestingly, this study showed that people practicing sports every day were characterized by a slightly higher percentage of depression and anxiety disorders than people who showed less regular physical activity. In the literature, you can come across the term addiction to physical exercise. It consists of regular, daily physical exercise regardless of the circumstances, also during illness, after an injury or when there are other health contraindications. Physical activity begins to dominate the addict’s life, which leads to the neglect of other areas of functioning, such as work, family life or social contact. In studies of a group of people practicing sports every day, after a break in exercise lasting more than a day, symptoms of withdrawal syndrome were found, the symptoms of which were anxiety, nervousness, irritability and depressed mood, which was dictated by changes in neurotransmitters [44]. In addition, too intense and too frequent training can be interpreted by the body as an excessive burden, generating chronic inflammation and initiating neurodegeneration [45]. 

Another group of analyzed factors was related to the use of psychoactive substances. According to the results of the study, the use of alcohol over three or more times a week was associated with an increased risk of anxiety disorders (26.5%). In this study, the influence of frequent alcohol use on the occurrence of depression has not been proven. The literature shows that alcohol dependence increases the incidence of depression almost fourfold, and anxiety disorders more than twofold. Among alcohol-dependent patients, 37% suffer from other mental disorders at the same time. This relationship is two-way, namely primary mental disorders may favor excessive drinking and the development of alcohol dependence, and alcohol abuse may induce or aggravate the occurrence of mental disorders. Drinking alcohol can also be an attempt to "self-medicate" anxiety or depression symptoms [46]. 

In the study group, anxiety and depressive disorders occurred significantly more often among smokers. The negative effects of cigarette smoking have been documented in many scientific studies [47]. Additionally, the coexistence of depressive symptoms in a smoker supports the habit of reaching for a cigarette [48]. One cohort study found that as exposure to higher doses of nicotine increased, the risk of depressive disorders increased, with women being more exposed at lower doses than men [49]. In addition, it is emphasized that people addicted to nicotine use marijuana and derivatives more often and with greater freedom [50]. With regard to stimulants other than tobacco and alcohol, which were included in this study, it should be emphasized that the percentage of people who confirmed their use was small and for marijuana it was 3.7% of the respondents, for stimulants 1.3%. In addition, the researchers in the questionnaire did not ask about the amount of substances and frequency of use, while being unable to assess the risk of addiction in the respondents. The use of marijuana has been confirmed mainly by young people, up to 29 years of age. There was no statistically significant difference in the assessment of the prevalence of anxiety and depression in this group. However, among the 22 people who confirmed the use of psychostimulants, the symptoms of anxiety disorders were significantly more frequent—36.4% vs. 16.3%. According to the results of the EZOP survey, 1.3% of respondents admitted to using psychoactive substances in Poland [3]. It is known from the literature that regular marijuana use is associated with an increased risk of depression [51]. The dependence of the occurrence of general sadness was demonstrated in 20–30% of people who smoked it [52]. In the Polish study of users of psychostimulants, higher values of the Beck Depression Scale were found in their group. The total value of points on the scale was higher, the more psychoactive substances the respondents used [53]. Many publications show that the use of amphetamine and methamphetamine and its derivatives may cause anxiety disorders of considerable intensity and uncontrolled impulsive behavior. The described symptoms may also occur in users of these substances during the period of abstinence [54]. It is estimated that the frequency of anxiety disorders among users of psychostimulants may be as high as 30.2% [55]. 

Among the subjects with somatic disease, anxiety disorders (23.3% vs. 13.2%) and depression (16.5% vs. 5.1%) were significantly more frequent than in healthy subjects. In this case, the relationship is bidirectional. In long-term studies, it was found that the presence of a depressive episode is associated with a much higher risk of developing somatic diseases [56], and vice versa, the presence of a somatic disease is a risk factor for depressive disorders, which significantly worsen the course of treatment and rehabilitation [57]. Pain, which is a symptom of many diseases, especially at severe stages, may be associated with the onset of a depressive or anxiety disorder [12,58]. In addition, it has been shown that the coexistence of anxiety disorders in patients with somatic disease increases the perceived somatic symptoms, while reducing the effectiveness of treatment [59].

This research showed that in the group of somatic patients, depression was slightly more frequent among people receiving regular treatment than in those who did not take medications permanently (17.8% vs. 11.1%). There are many reports that the inadequate treatment of somatic diseases carries a greater risk of depressive episodes. However, the observations made by the authors of this study may suggest that patients who do not take medications regularly may not feel any discomfort due to the coexistence of comorbidities. A typical example of this phenomenon is metabolic diseases such as arterial hypertension, diabetes, hyperlipidemia or gout, which at the beginning may not cause significant suffering to patients, and thus the incidence of depressive disorders in this group will also be lower. The feeling of being healthy strengthens the tendency to displace or downplay the disease, and it is known from the available analyses that compliance by patients leaves much to be desired [60]. 

Somatically burdened people who did not receive regular treatment confirmed the occurrence of anxiety disorders significantly more often compared to those who regularly took medication—34.3% vs. 20.7%. In the analysis of patients’ attitudes towards the diagnosis and treatment of the disease, there is a view that a significant reduction in the fear of finding oneself in the role of a sick person is achieved by an active attitude consisting of searching for information and actually relating it to one’s own situation, as well as focusing on controlling the state of affairs and overcoming the disease [61]. 

In the group of people using sleeping pills every day, depression was more than twice as common as in those untreated for insomnia. This difference, probably due to the small size of the group of people taking hypnotics, was not statistically significant. Analyzing the frequency of anxiety disorders, it was much higher in people taking hypnotics in general (43.8–55%) compared to the group of people not receiving treatment (13.8%). The literature shows that the most common form of sleep disturbance is insomnia. Its prevalence, depending on the research method adopted, ranges from 4.4% to 48%. It is known from Polish studies that the prevalence of insomnia is estimated at 50.5% [62]. Insomnia is three times more common in people diagnosed with depressive disorders. As a rule, it is a symptom of the current episode [63]. Additionally, sleep disorders increase the risk of the occurrence, recurrence and more severe course of depressive disorders [64] and may be a risk factor for anxiety disorders [65].

The frequency of use of over-the-counter medications or sedative supplements in the study population was directly proportional to the frequency of anxiety and depressive disorders. In the group of people using the above-mentioned preparations every day, anxiety disorders occurred almost four times as often and depressive disorders more than twice as often as in people who refused to use them. Every year, more and more new over-the-counter preparations are created. They are often the first link in the self-healing process of the studied disorders [66]. For many people, supplements, including herbal drugs, seem to be more natural and less harmful than synthetic psychotropic drugs. Usually, they are available without a prescription and do not require a visit to a specialist doctor, which theoretically is to protect users from stigma. Research conducted among people with anxiety and mood disorders has shown that nearly half of them use so-called complementary medicine [67]. Their use can often prolong the poor functioning of patients and delay the initiation of adequate treatment based on the principles of EBM (Evidence-Based Medicine) [68,69]. 

This publication is an example of a population study, the results of which enable the identification of new risk factors for the described disorders and the planning of changes in the scope of health policy, including targeted interventions.

## 5. Limitation 

Analyses of factors modifying the risk of anxiety disorders and depressive disorders were carried out thanks to a population study in the Żywiec district. Due to its demographic characteristics and compact geographic location, this area was selected as optimal for this type of research. A limitation, however, may be the structure of this region, the vast majority of which consists of rural areas. Therefore, much less data were obtained from people living in the city, which could result in a lack of significant differences in the incidence of anxiety disorders and depression depending on the place of residence.

People over 60 were the least numerous age group in the study. At the same time, in this group, the greatest number of respondents met the depression criteria. There is a chance that if the number of respondents in this age group was increased, the frequency of the examined disorders would be even greater. However, it can be assumed that elderly people who suffered from the tested symptoms were more likely to participate in the study, which could significantly inflate the results.

In the group of people who denied alcohol use, anxiety disorders were more frequently observed. The question about alcohol use was about the last month. It is difficult to estimate whether abstinence was forced by previous alcohol addiction, taking medications due to comorbidities or fear of addiction.

The too small size of the group of people using marijuana was probably the reason why smoking was not significantly influenced by anxiety and depression, which could be due to the reluctance of the respondents to disclose this information.

The HADS scale, which is a short and easy-to-fill diagnostic tool, detects depressive symptoms while not differentiating the severity of a depressive episode, which may lead to the overestimation of the percentage of respondents with depressive disorders. On the other hand, there is a risk that people with the greatest severity of symptoms were unable to complete the questionnaire on their own, which could lead to underestimating the scale of depression.

Due to the presence of more than one risk factor in one person, it is difficult to assess their independent influence on the occurrence of the analyzed disorders.

## 6. Conclusions

### 6.1. Concerning the Risk of Depressive Disorders

Factors increasing the risk of depressive disorders include:

- Being female;
- Age over 60;
- Being unemployed;
- Lower than secondary education;
- Performing mental work;
- Complete lack of physical activity and daily, intensive sports;
- Regular smoking of cigarettes;
- Chronic somatic diseases;
- Abuse of hypnotics and over-the-counter drugs with sedative potential.

In relation to depression, the protective effects were:

- Male gender;
- Age between 40 and 59;
- Higher education;
- Full-time employment or self-employment;
- Performing physical work.

The occurrence of depressive disorders was not influenced by:

- Marital status;
- Place of residence;
- Use of alcohol, marijuana and stimulants.

### 6.2. Concerning the Risk of Anxiety Disorders

Factors increasing the risk of anxiety disorders include:

- Belonging to the group of the unemployed, self-employed or retired;
- Lack of physical activity;
- Daily use of alcohol or complete abstinence;
- Regular smoking and the use of stimulants;
- The presence of somatic diseases;
- Over-the-counter drugs and drugs prescribed by a doctor to help with sleep.

The occurrence of anxiety disorders was significantly lower in the group of people who were:

- Employed full-time;
- Occasional alcohol users.

The presence of anxiety symptoms was not influenced by:

- The sex of respondents;
- Marital status;
- Living in a village or city;
- Level of education;
- Type of job;
- Marijuana use.

## Figures and Tables

**Table 1:** Characteristics of the study group—selected risk modifying factors.

Risk Modifying Factors	Study Group	
	*n*	%
Professional activity		
Full time	1044	62.9
Own business	120	7.2
Odd jobs	73	4.4
Studying	126	7.6
Unemployed	90	5.4
Pensioner	206	12.4
Type of job		
Physical work	707	42.6
Mental work	463	27.9
Unemployed	489	29.5
Physical activity		
Never	414	24.9
Occasional (2–3x/month)	725	43.7
Several times/week	406	24.5
Every day (regularly)	114	6.9
Frequency of drinking alcohol		
Not at all/last month	665	40.1
2–3 times/last month	588	35.4
1–2 times/week	285	17.2
3 or more times/week	121	7.3
Smoking		
Yes	329	19.8
No	1330	80.2
The use of cannabinoids		
Yes	62	3.7
No	1597	96.3
The use of psychostimulants		
Yes	22	1.3
No	1637	98.7
Presence and treatment of somatic diseases		
“I get sick, I take medications regularly”	455	27.4
“I get sick, I don’t take medications”	108	6.5
“I’m not sick”	1096	66.1
The use of hypnotics		
No	1513	91.2
Yes, occasionally	105	6.3
A few times a week	22	1.3
Every day	19	1.1
Use of sedative or hypnotic herbal medications or OTC dietary supplements		
No	1270	76.6
Yes, occasionally	299	18
A few times a week	47	2.8
Yes everyday	43	2.6

**Table 2:** Risk modifying factors and scores on the HADS-D and HADS-A subscales.

Risk Modifying Factor	HADS-D1		HADS-D2		HADS-D3		*p*-Value	HADS-A1		HADS-A2		HADS-A3		*p*-Value
	*n*	%	*n*	%	*n*	%		*n*	%	*n*	%	*n*	%	
Sex														
Female	578	61.9	181	19.4	175	18.7	<0.01	736	78.8	113	12.1	85	9.1	NS *
Male	566	78.1	94	13.0	65	8.9	<0.01	539	74.3	85	11.7	101	13.9	NS
Age range														
18–39 years	540	84.5	78	12.2	21	3.3	<0.01	444	69.5	99	15.5	96	15	NS
40–59 years	656	84.2	79	10.1	44	5.7	<0.01	522	67	128	16.4	129	16.6	NS
≥60 years	107	44.3	50	20.8	84	34.9	<0.01	152	63	39	16.2	50	20.8	NS
Marital status														
Single	394	79.6	64	12.9	37	7.5	NS	335	67.7	73	14.8	87	17.6	NS
Steady relationship	909	78.1	143	12.3	112	9.6	NS	783	67.3	193	16.6	188	16.2	NS
Place of residence														
City	398	81.1	59	12	34	6.9	NS	337	68.6	81	16.5	73	14.9	NS
Village	905	77.5	148	12.7	115	9.9	NS	781	66.9	185	15.8	202	17.3	NS
Education														
Basic	100	66.2	27	17.9	24	15.9	<0.01	93	61.6	25	16.6	33	21.9	NS
Vocational	302	74.8	50	12.4	52	12.8	<0.01	257	63.6	64	15.8	83	20.5	NS
Medium	507	81.1	73	11.7	45	7.2	<0.01	425	68	110	17.6	90	14.4	NS
Higher (Bachelor’s D.)	125	84.5	13	8.8	10	6.8	<0.01	108	72.9	19	12.8	21	14.2	NS
Higher (Master’s D.)	269	81.3	44	13.3	18	5.4	<0.01	235	71	48	14.5	48	14.5	NS
Professional activity														
Full time	930	89.1	85	8.1	29	2.8	<0.01	749	71.7	158	15.1	137	13.1	<0.01
Own business	102	85.0	15	12.5	3	2.5	<0.01	75	62.5	18	15	27	22.5	<0.01
Odd jobs	69	94.5	1	1.4	3	4.1	<0.01	49	67.1	15	20.6	9	12.3	<0.01
Studying	93	73.8	28	22.2	5	4.0	<0.01	81	64.3	21	16.7	24	19	<0.01
Unemployed	63	70	23	25.6	4	4.4	<0.01	49	54.4	15	16.7	26	28.9	<0.01
Pensioner	46	22.3	55	26.7	105	51	<0.01	115	55.8	39	18.9	52	25.2	<0.01
Type of job														
Physical	678	97.7	13	1.9	3	0.4	<0.01	482	69.5	113	16.3	99	14.3	NS
Mental	374	80.8	64	13.8	25	5.4	<0.01	325	70.2	64	13.8	64	13.8	NS
Not working	12	92.3	1	7.7	0	0	<0.01	8	61.5	0	0	5	38.5	NS
Physical activity														
Never	294	71	53	12.8	67	16.2	<0.01	230	55.6	80	19.3	104	25.1	<0.01
Occasional (2–3x/month)	611	84.3	79	10.9	35	4.8	<0.01	501	69.1	122	16.8	102	14.1	<0.01
Several times/week	314	77.3	59	14.5	33	8.1	<0.01	299	73.7	55	13.6	52	12.8	<0.01
Every day (regularly)	84	73.7	16	14	14	12.3	<0.01	88	77.2	9	7.9	17	14.9	<0.01
Drinking alcohol														
Not at all/last month	515	77.4	83	12.5	67	10.1	NS	448	67.4	96	14.4	121	18.2	<0.01
2–3 times/last month	474	80.6	70	11.9	44	7.5	NS	402	68.4	110	18.7	76	12.9	<0.01
1–2 times/week	223	78.3	37	12.9	25	8.8	NS	195	68.4	44	15.4	46	16.1	<0.01
3 or more times/week	91	75.2	17	14.1	13	10.7	NS	73	60.3	16	13.2	32	26.5	<0.01
Smoking														
Yes	261	79.3	28	8.5	40	12.2	<0.01	195	59.3	59	17.9	75	22.8	<0.01
No	1042	78	179	13.5	109	8.2	<0.01	923	69.4	207	15.6	200	15	<0.01
The use of cannabinoids														
Yes	55	88.7	5	8.1	2	3.2	NS	37	59.7	13	21	12	19.4	NS
No	1248	78	202	12.7	147	9.2	NS	1081	68	253	15.8	263	16.5	NS
The use of psychostimulants														
Yes	20	90.9	1	4.6	1	4.6	NS	13	59.1	1	4.6	8	36.4	<0.05
No	1281	78	205	12.6	148	9.1	NS	1103	68	265	16.2	266	16.3	<0.05
Presence and treatment of somatic diseases														
“I get sick, I take medications regularly”	306	67.3	68	15	81	17.8	<0.01	268	58.9	93	20.4	94	20.7	<0.01
“I get sick, I don’t take medications”	82	75.9	14	13	12	11.1	<0.01	52	48.2	19	17.6	37	34.3	<0.01
“I’m not sick”	915	83.5	125	11.4	56	5.1	<0.01	798	72.8	154	14.1	144	13.2	<0.01
The use of drugs with a hypnotic effect														
No	1203	80	181	12	129	8.5	NS	1059	70	246	16.3	208	13.8	<0.01
Yes, occasionally	75	71.4	17	16.2	13	12.4	NS	44	41.9	15	14.3	46	43.8	<0.01
A few times a week	14	63.6	5	22.7	3	13.6	NS	6	27.3	4	18.2	12	55	<0.01
Every day	11	57.9	4	21.1	4	21.1	NS	9	47.4	1	5.3	9	47.4	<0.01
The use of OTC drugs/supplements with a sedative or hypnotic effect														
No	1023	81	150	11.8	97	10.7	<0.01	925	72.8	195	15.4	150	11.8	<0.01
Yes, occasionally	225	75.3	42	14.1	32	10.7	<0.01	157	52.5	51	17.1	91	30.4	<0.01
A few times a week	29	61.7	8	17	10	21.3	<0.01	19	40.4	11	23.4	17	36.2	<0.01
Yes everyday	26	60.5	7	16.3	10	23.3	<0.01	17	39.5	9	20.9	17	39.5	<0.01

NS *—statistically insignificant.

## Data Availability

The data presented in this study are available on request from the last author.
